# Attitudes of publics who are unwilling to donate DNA data for research

**DOI:** 10.1016/j.ejmg.2018.11.014

**Published:** 2019-05

**Authors:** Anna Middleton, Richard Milne, Adrian Thorogood, Erika Kleiderman, Emilia Niemiec, Barbara Prainsack, Lauren Farley, Paul Bevan, Claire Steed, James Smith, Danya Vears, Jerome Atutornu, Heidi C. Howard, Katherine I. Morley

**Affiliations:** a Society and Ethics Research, Connecting Science, Wellcome Genome Campus, Cambridge, UK; b Faculty of Education, University of Cambridge, Cambridge, UK; c Institute of Public Health, University of Cambridge, Cambridge, UK; d Centre of Genomics and Policy, McGill University, Montreal, Quebec, Canada; e Centre for Research Ethics and Bioethics, Uppsala University, Uppsala, Sweden; f Department of Political Science, University of Vienna, Austria; g Department of Global Health & Social Medicine, King's College London, UK; h Web Team, Wellcome Sanger Institute, Wellcome Genome Campus, Cambridge, UK; i Center for Biomedical Ethics and Law, Department of Public Health and Primary Care, KU Leuven, Leuven, Belgium; j Melbourne Law School, The University of Melbourne, Melbourne, Australia; k Biomedical Ethics Research Group, Murdoch Children's Research Institute, Parkville, Australia; l Institute of Psychiatry, Psychology, and Neuroscience, King's College London, London, UK; m Centre for Epidemiology and Biostatistics, Melbourne School of Global and Population Health, The University of Melbourne, Melbourne, Australia; n School of Health Sciences, University of Suffolk, Ipswich, UK

## Abstract

With the use of genetic technology, researchers have the potential to inform medical diagnoses and treatment in actionable ways. Accurate variant interpretation is a necessary condition for the utility of genetic technology to unfold. This relies on the ability to access large genomic datasets so that comparisons can be made between variants of interest. This can only be successful if DNA and medical data are donated by large numbers of people to ‘research’, including clinical, non-profit and for-profit research initiatives, in order to be accessed by scientists and clinicians worldwide.

The objective of the ‘Your DNA, Your Say’ global survey is to explore public attitudes, values and opinions towards willingness to donate and concerns regarding the donation of one's personal data for use by others. Using a representative sample of 8967 English-speaking publics from the UK, the USA, Canada and Australia, we explore the characteristics of people who are unwilling (n = 1426) to donate their DNA and medical information, together with an exploration of their reasons. Understanding this perspective is important for making sense of the interaction between science and society. It also helps to focus engagement initiatives on the issues of concern to some publics.

## 1 Introduction

### 1.1 Large genomic datasets underpin genomic medicine

In order to fully realise the potential of genomic medicine to predict, diagnose, manage and treat genetic disease, clinical scientists routinely access de-identified genomic datasets containing DNA and medical information from large numbers of people. Projects such as the Million European Genomes Alliance plan to link electronic patient records with genomic sequencing results from 1 million people across Europe in order to meet this need for very large datasets with both genotype and phenotype information (European Commission, 2018)*.*

There has been wide support (Boycott et al., 2017; Budin-Ljosne et al., 2014) for genomic data sharing, together with a call for practical and ethical solutions to connect genomic databases and make them more accessible for clinical and research purposes (Thompson et al., 2014). Such health-related data are sensitive and may be potentially misused (e.g. discrimination) or used in ways not supported by the data contributors (Sterckx et al., 2016). Consequently, scholars have called for consistent ethical and legal frameworks that enable access across institutional and national jurisdictions that respect and protect individuals who have contributed data (Knoppers et al., 2014; Borry et al., 2018). Such regulatory frameworks in turn aim to meet the needs of people who currently refuse to participate in genetic research out of fear for genetic discrimination (Wauters and Van Hoyweghen, 2016).

To determine whether a variant is consistently linked to a particular phenotype, datasets should ideally contain information from people of varying ages, disease status, health and ethnicity. However, many existing genomic databases have limitations in terms of the population they represent, with the vast majority of studies focussed primarily on European populations (Popejoy and Fullerton, 2016; Landry et al., 2018). In order to meet the demand for larger numbers and more diversity, millions of people globally will need to donate and share their data (Middleton, 2018; Birney et al., 2017). There have thus been calls to encourage the donation of data which includes a broader population as well as disease status, in order to support the equitable delivery of genomic medicine (Sirisena and Dissanayake, 2017).

In this manuscript, we use the term ‘data donation’ to refer to the decision an individual makes to contribute their genomic data to a database that can be accessed by researchers or clinicians. The act of ‘data donation’ assumes that these data will be subsequently accessed by and shared with others for clinical and research purposes. Opportunities to donate DNA and/or medical data arise when individuals undergo genetic testing (Wright et al., 2017), store samples in biobanks (Small, O'Donnell, and Damrauer, 2018), participate in clinical research (Auffray et al., 2016) or when they donate their blood to blood banks (Hartling et al., 2015). Understanding what motivates and de-motivates data donation is pivotal to creating appropriately designed engagement, dialogue strategies and regulatory frameworks about genomic medicine and research.

### 1.2 Attitudes towards data donation

Empirical research on public attitudes to data sharing tells us that, broadly speaking, publics are willing to donate and share their health data with researchers (Weitzman et al., 2012). One driver of this is the perceived use of data and expectations of data users, as many participants see their contribution to research as helping towards the ‘public good’ (Dixon-Woods and Tarrant, 2009). In contrast, participants' view of data donation becomes less positive when they are not consulted on the uses of their data (McCormack et al., 2016) or believe that their donation will primarily lead to big profits for commercial companies, without creating obvious public value (Trinidad et al., 2010). Research from Wellcome has shown participation in research and data sharing are viewed as more acceptable when care and attention is paid to explaining the necessary partnerships between industry and healthcare (Wellcome , 2016). In addition, previous survey research, primarily in the USA, has suggested that there may be important differences in the characteristics of those willing to donate their genomic data to research, particularly associated with education, race, religiosity and levels of perceived benefit and concern (Sanderson et al., 2017; Shabani et al., 2014).

In this paper, we focus on the characteristics and reasoning of people who are unwilling to donate their DNA and medical data to research using findings from the ‘Your DNA, Your Say’ international survey. The ‘Your DNA, Your Say’ survey is part of a global project that has been translated into several languages, including: Russian, Polish, Portuguese, Spanish, German, Icelandic, Swedish, French, Japanese, Urdu, Arabic, and Italian with plans to translate into Hindi, Mandarin, Zulu, Twi and Ewe. Once global recruitment is completed, we will perform a between-country meta-analysis of attitudes, which will be described separately. Here we present here data from the English-speaking participants (for whom recruitment has finished). The survey was developed in collaboration with the Participant Values task team of the Global Alliance for Genomics and Health, a public-private consortium developing policy frames and technical standards for the exchange of genomic and health-related data.

## 2 Methods

A more detailed description of the methodological rationale for the study, design (and limitations), recruitment strategy, and process of data collection have been published separately (Middleton et al., 2018), as has a review of the context and background to this project (Middleton, 2018).

### 2.1 Sample

Using a market research company, ResearchNow, we collected completed surveys from publics in the USA, Canada, United Kingdom (UK) and Australia (n = 8967). Participants were paid a small financial reward (<£1) for participating and due to the nature of recruitment there are no details on non-response rate. Our participant samples are ‘representative’, according to recent census data, of populations in Canada, the USA, the UK and Australia in terms of age and gender. However, as we did not specifically aim to recruit a ‘representative’ sample according to self-reported ethnicity, the ethnic diversity of our sample cannot be considered ‘ethnically representative’. We found only small variation in between-country analysis and have corrected for this in the modelling; thus, in this paper we have chosen not to focus specifically on differences between participant attitudes from the UK, the USA, Canada, and Australia but to explore the collective profiles of those participants, across countries.

### 2.2 Measures

Our cross-sectional, exploratory online survey can be accessed from www.YourDNAYourSay.org. It contains 29 questions and piloting showed it took approximately 15–20 min to complete.

#### 2.2.1 Donating DNA and medical information

Throughout the survey, participants were asked whether they would donate their “anonymous''^1^ DNA and medical information for use by others in research. We asked participants to distinguish who they would allow to use their data, (a) medical doctors; (b) non-profit researchers; (c) for-profit researchers. Participants were classified as *willing to donate* if they answered “yes” to at least one of these questions, *unwilling to donate* if they answered “no” to all three, and *unsure* if they answered “unsure” to all three. We will publish separately on the profile of those who were more accepting of data donation (Middleton et al., under review) and on the distinctions participants make between users of their data.

#### 2.2.2 Sociodemographics

Age was collected in ten-year categories from age 16 onwards, but due to the lower number of responses in younger and older age categories these were collapsed into three categories of “30 years and under”, “31–50“, and “51 years and older'' for analysis. Whether participants had children was determined by a “Yes'' or “No'' answer without specifying whether the children were biological or not. Relationship status was collected as “Divorced'', “Separated'', “Single'', “Widowed'', “Married/civil partnership/living together'', but all categories apart from the latter were collapsed for analyses.

We piloted how best to collect ethnicity data, starting with the categories provided in the UK Census survey and adapting these, based on feedback from pilot participants involved in survey development. The resultant ethnicity question in the final survey thus asked participants to self-identify as (1) White, (2) Afro-European/African American, Black (3) Hispanic (4) South Asian, Indian, Pakistani (5) East Asian Chinese, Japanese (6) Arabic, Central Asian (7) Other (Table 1). Participants could also choose not to answer this question at all. In the analysis, due to the low number of participants who self-identified as a member of a group other than “White” (less than 10% of the sample for each country), these were collapsed into a single “Non-White” category for analysis. Highest level of education was categorised as “Tertiary'', “Secondary'', “Primary'' or “Other'' based on structured responses and also free-text descriptions of educational qualifications. This was collapsed to a binary indicator of tertiary education for multivariable analyses. Religiosity was determined by participant response to the question “Independent of whether you attend religious services or not, would you say you are … ?'' with options “A religious person'' or “Not a religious person''.

**Table 1:** Sample description (n = 8961). Participants willing, unwilling and unsure about donating DNA and/or medical information to research (including to medical doctors, non-profit and for-profit researchers) associated with familiarity about genetics and demographic variables. P-values for χ^2^ tests (excluding missing data) are shown.

Variable	Categories	Total (n = 8961)		Willing (n = 6073)		Unwilling (n = 1426)		Unsure (n = 1462)		P
		N	%	N	%	N	%	N	%	
Genetics knowledge	Unfamiliar	5004	55.8	3036	50	918	64.4	1050	71.8	<0.0001
	Familiar	2786	31.1	2052	33.8	407	28.5	327	22.4	
	Personal	1170	13.1	985	16.2	100	7	85	5.8	
	Missing	1	0	0	0	1	0.1	0	0	
Age	30 and under	2090	23.3	1493	24.6	270	18.9	327	22.4	<0.0001
	31–40	2046	22.8	1406	23.2	310	21.7	330	22.6	
	41–50	1569	17.5	988	16.3	272	19.1	309	21.1	
	51–60	1588	17.7	1011	16.6	297	20.8	280	19.2	
	Over 60	1664	18.6	1172	19.3	277	19.4	215	14.7	
	Missing	4	0	3	0	0	0	1	0.1	
Gender	Female	4328	48.3	2895	47.7	631	44.2	802	54.9	<0.0001
	Male	4573	51	3154	51.9	780	54.7	639	43.7	
	Missing	60	0.7	24	0.4	15	1.1	21	1.4	
Children	No	3695	41.2	2445	40.3	641	45	609	41.7	0.001
	Yes	5111	57	3556	58.6	743	52.1	812	55.5	
	Missing	155	1.7	72	1.2	42	2.9	41	2.8	
Education	Tertiary	5172	57.7	3664	60.3	759	53.2	749	51.2	<0.0001
	Secondary	3009	33.6	1943	32	520	36.5	546	37.3	
	Primary	551	6.1	331	5.5	103	7.2	117	8	
	Other	224	2.5	131	2.2	44	3.1	49	3.4	
	Missing	5	0.1	4	0.1	0	0	1	0.1	
Country	United Kingdom	3316	37	2257	37.2	486	34.1	573	39.2	0.006
	United States	1992	22.2	1366	22.5	334	23.4	292	20	
	Canada	2251	25.1	1544	25.4	349	24.5	358	24.5	
	Australia	1402	15.6	906	14.9	257	18	239	16.3	
Ethnicity	Afro-European, African American, Black	322	3.6	211	3.5	56	3.9	55	3.8	0.088
	Asian	660	7.4	422	6.9	117	8.2	121	8.3	
	Hispanic	139	1.6	85	1.4	27	1.9	27	1.8	
	Other	193	2.2	121	2	36	2.5	36	2.5	
	White	7538	84.1	5186	85.4	1150	80.6	1202	82.2	
	Missing	109	1.2	48	0.8	40	2.8	21	1.4	
Religiosity	Not a religious person	5608	62.6	3695	60.8	923	64.7	990	67.7	<0.0001
	A religious person	3348	37.4	2374	39.1	503	35.3	471	32.2	
	Missing	5	0.1	4	0.1	0	0	1	0.1	
Relationship	Married/civil partnership/living together	5564	62.1	3847	63.3	829	58.1	888	60.7	0.001
	Divorced/Single/Widowed	3392	37.9	2222	36.6	597	41.9	573	39.2	
	Missing	5	0.1	4	0.1	0	0	1	0.1	

#### 2.2.3 Genetics experience

Genetics experience was derived from two variables: “Are you familiar with DNA, genetics, or genomics?'' If a respondent chose the answer: “I'm familiar through my work, personal interests or family/medical history'', they could further specify. Participants were categorised as having “Personal'' experience of genetics if they said they were familiar with DNA/genetics/genomics and that familiarity was due to either having a genetic condition in their family, or through their work (e.g. genetic health professional or genetic researcher). Participants without this experience were categorised as “Familiar'' or “Unfamiliar'' based on their response to the first question.

#### 2.2.4 Potential for harm

Participants were asked a single question regarding harms associated with linking personally identifying information to their DNA data: “If someone linked your name, address and phone number to it, do you think you could be harmed in any way from this?'' Response options were “Yes'', “No'', “I'm not sure'' with the latter two categories collapsed for analysis.

#### 2.2.5 Concerns about specific harms

Participants were presented with a list of hypothetical harms that could occur in relation to DNA information and asked to indicate which three of these concerned them the most. The list of hypothetical harms was based on pilot work, the academic literature and experience of the authors who designed the survey. The list of hypothetical harms presented to participants was:

• My friends potentially knowing something about me that I hadn't chosen to tell them
• My family potentially knowing something about me that I hadn't chosen to tell them
• My government potentially knowing something about me that I hadn't chosen to tell them
• Police potentially knowing something about me that I hadn't chosen to tell them
• Marketing companies targeting me to sell me products
• Being stigmatised and labelled in some way online
• Being cloned
• My DNA being copied and then planted at the scene of a crime
• Health or life insurance companies using the information to discriminate against me
• Employers using the information to discriminate against me
• Upsetting my genetic relatives
• Ethnic identification and racial discrimination

#### 2.2.6 Factors affecting the decision to donate

Participants were asked to identify what factors would influence their decision to donate their DNA and medical information. They were asked to select from the following list (multiple selections possible):

• Whether my identifying information (age, sex, etc.) will be included or not
• Who has control over access to my information
• What sorts of research my information could be used in
• The potential risks and benefits of making a donation
• How the researcher might benefit from accessing my information
• What sort of researchers are likely to access my information
• How I might be acknowledged for my contribution to scientific knowledge
• If the researchers were going to make money with the results
• Whether I would have access to the DNA readout generated by researchers
• If I can participate in the governance of data access
• How I will be assisted if there is a data breach

### 2.3 Statistical analysis

Sample characteristics were summarised using standard descriptive statistics, and bivariate relationships were evaluated using χ^2^ tests as all variables were categorical. Importance of p-values was considered in the context of multiple testing. The multivariable analysis of participant characteristics associated with donation preference was conducted using a multinomial logistic regression model with donation preference as the outcome variable. A complete-case sample was used. We have previously used multi-level models to analyse these data (Middleton et al., under review) but comparison of model fit showed that a multi-level model was not necessary for donation preference. Familiarity with genetics, age, gender, ethnicity, country of residence, marital status, having children, education level, and religiosity were included as covariates. As this model was explanatory rather than predictive, no variable selection methods were used and the full model is presented.

## 3 Results

### 3.1 Attitudes towards donation

In this sample, 15.9% of participants (n = 1426) reported that they were unwilling to donate their DNA and medical information to medical doctors, non-profit researchers, or for-profit researchers (with the explained assumption that these professionals would then access and share this data with others in their specific field). A further 16.3% (n = 1462) were unsure in all cases. The majority (67.7%; n = 6073) were willing to donate in at least one scenario (this latter group are explored in more depth elsewhere, Middleton et al., under review). Data on this topic were missing for 6 participants, who were excluded from further analyses.

Compared to those who were willing to donate (Table 2), those who were unsure or unwilling had substantially lower odds of being familiar with, or having a personal experience of, genetics/genomics. Both of these groups also had lower odds of being aged 30 and under, and higher odds of not having a tertiary-level qualification and of self-identifying as a member of an ethnic group other than White.

**Table 2:** Multinomial logistic regression result for views on donation, with willing to donate as reference category (n = 8703), associated with familiarity about genetics and demographic data. OR indicates odds ratio; LCI indicates lower 95% confidence interval; UCI indicates upper 95% confidence interval.

Variable	Category	Unwilling				Unsure			
		OR	LCI	UCI	P	OR	LCI	UCI	P
Genetics knowledge	Unfamiliar	ref.				ref.			
	Familiar	0.66	0.58	0.76	<0.0001	0.5	0.43	0.58	<0.0001
	Personal	0.35	0.28	0.43	<0.0001	0.25	0.2	0.32	<0.0001
Age	Over 50	ref.				ref.			
	31–50	0.93	0.81	1.07	0.34	1.2	1.04	1.38	0.01
	30 and under	0.64	0.54	0.77	<0.0001	0.9	0.75	1.07	0.22
Gender	Female	ref.				ref.			
	Male	1.12	0.99	1.27	0.06	0.74	0.66	0.84	<0.0001
Children	No	ref.				ref.			
	Yes	0.74	0.65	0.84	<0.0001	0.92	0.81	1.05	0.20
Tertiary education	Yes	ref.				ref.			
	No	1.26	1.11	1.43	0.0003	1.31	1.16	1.49	<0.0001
Country	United Kingdom	ref.				ref.			
	United States	1.37	1.15	1.62	0.0003	1.08	0.91	1.29	0.36
	Canada	1.09	0.93	1.28	0.29	1.08	0.92	1.27	0.33
	Australia	1.28	1.07	1.53	0.008	1.09	0.91	1.3	0.34
Ethnicity	White	ref.				ref.			
	Non-White	1.37	1.15	1.62	0.0004	1.35	1.14	1.61	0.0005
Religious person	No	ref.				ref.			
	Yes	0.87	0.76	0.99	0.03	0.78	0.69	0.9	0.0003

Table 2 also shows people who were unwilling to donate had a different sociodemographic profile than those who were unsure. Specifically, those unwilling and unsure about donating differed in relation to gender, having children, country of residence, and religiosity. Compared to those who were willing to donate, those who were unsure had much lower odds of being male (odds ratio (OR) 0.74; 95% confidence interval (CI) 0.66–0.84; p < 0.0001) whereas there was no gender difference between those who were and were not willing to donate. There was no difference between those people who were willing to donate and those who were unsure in terms of having children or country of residence, but these factors were strongly associated with being unwilling to donate; unwilling participants had much lower odds of having children (OR 0.74; 95%CI 0.65–0.84; p < 0.0001), and greater odds of residing in the USA or Australia (OR 1.37; 95%CI 1.15–1.62; p < 0.0001 and OR 1.28; 95%CI 1.07–1.53; p = 0.008 respectively). Those unsure about donating had lower odds of being religious (OR 0.78; 95%CI 0.69–0.90; p = 0.003), but there was no substantial difference between those who were and were not willing to donate in terms of religiosity. Put simply, those who were unsure about data donation were more likely to be female, to have children, and to reside in the USA or Australia, but less likely to be religious than those who were unwilling to donate data (both compared to those willing to donate their data).

### 3.2 Perceptions of harms arising from linking personal and DNA/medical information

A similar percentage of participants in the willing and unwilling to donate groups (45.1% and 45.6% respectively) believed that linking their DNA and medical information to their personal details could result in personal harm, whereas only 27.8% of unsure participants agreed with the statement (Table 3). This reflects the fact that those participants who were unsure overall were also more likely to be unsure about the potential for data linkage to result in harm, and thus less likely to agree with the statement.

**Table 3:** Numbers and percentages of participants in each donation group (i) agreeing that linkage of personal information to DNA could result in harm; (ii) endorsing individual potential harms. P-values for χ^2^ tests are shown.

Variable	Total		Willing		Unwilling		Unsure		P
	N	%	N	%	N	%	N	%	
Agreement to “*If someone linked your name, address and phone number to [your DNA], do you think you could be harmed in any way from this?”*	3816	42.6	2767	45.6	643	45.1	406	27.8	<0.0001

My DNA being copied and then planted at the scene of a crime	4050	45.2	2741	45.1	645	45.2	664	45.4	0.981
My family potentially knowing something about me that I hadn't chosen to tell them	1871	20.9	1311	21.6	277	19.4	283	19.4	0.057
My friends potentially knowing something about me that I hadn't chosen to tell them	1981	22.1	1368	22.5	277	19.4	336	23	0.027
Employers using the information to discriminate against me	2131	23.8	1503	24.7	306	21.5	322	22	0.007
Health or life insurance companies using the information to discriminate against me	3333	37.2	2379	39.2	480	33.7	474	32.4	<0.0001
Marketing companies targeting me to sell me products	3139	35	2230	36.7	438	30.7	471	32.2	<0.0001
My government potentially knowing something about me that I hadn't chosen to tell them	2882	32.2	1853	30.5	574	40.3	455	31.1	<0.0001
Police potentially knowing something about me that I hadn't chosen to tell them	1630	18.2	1042	17.2	339	23.8	249	17	<0.0001
Being stigmatised and labelled in some way online	1941	21.7	1362	22.4	257	18	322	22	0.001
Being cloned	2510	28	1556	25.6	453	31.8	501	34.3	<0.0001
Upsetting my genetic relatives (because my DNA information is similar to their DNA information)	1306	14.6	809	13.3	218	15.3	279	19.1	<0.0001

**Fig. 1:**
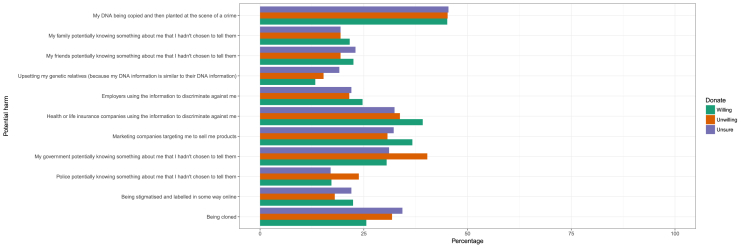
Hypothetical harms arising from linking personally identifiable information (name, address etc) to DNA information. Participants were asked to rate if they were concerned about these and then stratified according to willingness to donate DNA and/or medical data to research (including clinical, non-profit, for-profit research).

The potential harm identified most frequently by participants was “My DNA being copied and then planted at the scene of a crime”; 45% of each group endorsed this (see Table 3; Fig. 1). The three groups were also similar in terms of concern regarding family and friends knowing something about them, and employers using the information to discriminate against them. However, the three groups differed in relation to other concerns about potential harms. The other harms most frequently endorsed by the willing-to-donate group were: “Health or life insurance companies using the information to discriminate against me” (39.2%) and “Marketing companies targeting me to sell products” (36.7%).

The unwilling-to-donate group were also concerned about insurance discrimination (33.7%), and marketing uses of data (30.7%). However, they were more concerned about “My government potentially knowing something about me that I hadn't chosen to tell them” (40.3%). This was a higher percentage than those in the willing-to-donate and unsure groups (30.5% and 31.1% respectively; χ^2^ = 51.08; df = 2; p < 0.0001). This group was also more likely to be concerned about “Police potentially knowing something about me that I hadn't chosen to tell them” (23.8% compared to 17% of the other groups; χ^2^ = 35.53; df = 2; p < 0.0001).

### 3.3 Factors influencing the decision to donate DNA/medical information

**Fig. 2:**
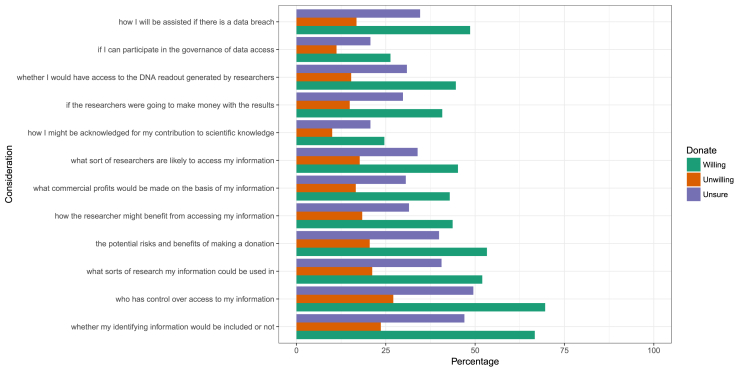
Considerations that might affect willingness to donate DNA and/or medical information, stratified by reported willingness to donate.

There were substantial differences between the three donation groups for all the factors influencing donation that participants were asked to explore (see Table 4; Fig. 2). However, the differences all followed the same pattern: participants who were unwilling to donate were proportionally less likely to identify a factor as influencing their decision to donate than those who were willing to donate.

**Table 4:** Numbers and percentages of participants in each donation group endorsing particular considerations regarding donation. P-values for χ^2^ tests are shown.

Variable	Total		Willing		Unwilling		Unsure		P
	N	%	N	%	N	%	N	%	
whether my identifying information	5076	56.6	4053	66.7	336	23.6	687	47	<0.0001
who has control over access to my information	5333	59.5	4224	69.6	386	27.1	723	49.5	<0.0001
what sorts of research my information could be used in	4056	45.3	3159	52	303	21.2	594	40.6	<0.0001
the potential risks and benefits of making a donation	4110	45.9	3234	53.3	293	20.5	583	39.9	<0.0001
how the researcher might benefit from accessing my information	3378	37.7	2656	43.7	262	18.4	460	31.5	<0.0001
what commercial profits would be made on the basis of my information	3289	36.7	2605	42.9	237	16.6	447	30.6	<0.0001
what sort of researchers are likely to access my information	3497	39	2748	45.2	253	17.7	496	33.9	<0.0001
how I might be acknowledged for my contribution to scientific knowledge	1939	21.6	1494	24.6	143	10	302	20.7	<0.0001
if the researchers were going to make money with the results	3126	34.9	2477	40.8	213	14.9	436	29.8	<0.0001
whether I would have access to the DNA readout generated by researchers	3381	37.7	2711	44.6	218	15.3	452	30.9	<0.0001
if I can participate in the governance of data access	2062	23	1600	26.3	160	11.2	302	20.7	<0.0001
how I will be assisted if there is a data breach	3697	41.3	2951	48.6	240	16.8	506	34.6	<0.0001

The factor that unwilling-to-donate participants most commonly identified as influencing their decision to donate was the question of who would control access to their information (27%), followed by whether identifying information would be included or not (24%). Participants were least likely to be influenced in their decision by whether they would be acknowledged for their contribution (10%) and whether they would be able participate in the governance of data access (11%).

## 4 Discussion

The focus of this paper is on the profile and characteristics of those participants who said they were unwilling to donate their DNA and medical data to be shared in clinical and scientific research. Our results show those members of the public who said they were unwilling to donate their DNA and medical data had a broad demographic profile. They were more likely to be older (men or women), without children, with lower levels of education, to be from the USA or Australia and to have self-reported ethnicity as any of the groups other than White. Along with those who were ‘unsure about donation’, those who were unwilling to donate were a group with little reported familiarity with DNA, genetics and genomics. The key question arising from the results is why this particular profile might be associated with being unwilling to donate one's data.

A first explanation would be that this group are more concerned than others about the overall harms associated with re-identification. Our data suggests this is not the case. The proportion who thought it was possible that they could be harmed was not greater amongst participants unwilling to donate than among those who were willing. Importantly, however, the types of concerns were different amongst this group. Specifically, in comparison with those who were willing to donate, the unwilling group were more likely to say that they were worried about the government or police knowing something about them that they had not chosen to share. They were less likely to identify harms associated with insurance and marketing uses of data.

These data suggest that increasing familiarity with DNA, genetics and genomics may not be enough to convert someone who is unwilling about donation to someone more willing to donate. Familiarity is a key difference between the otherwise similar profiles (in terms of all variables we measured) of those who are ‘unsure about donation’ and those who are willing to donate. Consequently, increasing familiarity may result in the current ‘unsures’ potentially converting to a ‘yes’ to donation. However, we are less confident about this prediction for those currently ‘unwilling to donate’. This group differ across variables from those who are willing to donate, both in terms of the potential harms they perceive and the factors which influence their decision to donate. Rather than familiarity, a key feature of this group appears to be unease with systems of legal and political authority, notably governments and the police. This may explain the lack of success of legal controls in limiting concerns about genetic discrimination (Wauters and Van Hoyweghen, 2016) and suggests the potential limitations of approaches to dialogue which do not take such concerns into account.

Whilst our research study did not set out to explore attitudes specifically from particular ethnic groups (although between-country attitudes will be explored) we have found that those who self-identify as a member of an ethnic group other than White have higher odds of being unwilling to donate their DNA or health data. The combination of concerns about government and police use of data with potential ethnic differences in the data echoes previous research which has reported concern that genetic results could be used to racially discriminate (Goldenberg et al., 2011). We all have, irrespective of our ethnic or racial background, a right to protection against discrimination. However, fear of discrimination is very significant for some (Wauters and Van Hoyweghen, 2016). Ethnic minority groups including African American/European Black and Asian groups have at times been reported to have a difficult relationship with genomics services (Mathew et al., 2017), the reasons for which are complex. Within the context of data donation for the assessment of disease risk (such as cancer) the perception of stigma (the notion of being at risk) and taboo could contribute to unwillingness to donate among ethnic minority groups along with low level of knowledge and awareness of familial cancer risk (Hann et al., 2017; Allford et al., 2014). Also, the aniticipated impact on minority communities could explain reluctance to donate; such as the stratification of society into people with “good” and “bad” genes leading to genomic medicine largely benefiting a privileged few (Bentley et al., 2017). This literature emphasises the importance of previous ethical injustices within medical research (Underwood et al., 2013) and fears of discrimination (Buseh et al., 2013) which provide potential causes for mistrust (Buseh et al., 2014) and fear (Catz et al., 2005). Steps should be taken to acknowledge and address these very real fears so as not to perpetuate perceptions of discrimination and persecution; policy makers have begun to explore practical steps to do this, including advocating the need for cultural literacy amongst geneticists, and promotion of evidence based ethical engagement strategies (Claw et al., 2018; Staunton et al., 2018).

Before deciding whether to offer one's DNA and medical data to be used in research, individuals would benefit from access to clear information about the risks and benefits, as well as details of the types of research for which the data will be used. Research and clinical programmes need to be more transparent about how they collect, store, process, and share data, as well as how they safeguard data against potential breaches and how such breaches will be dealt with if they occur. However, the practice of responsible genomic research also needs be accompanied by a public dialogue about the implications of genomic testing and the use of results. Even if we are not personally undergoing genetic testing, it is increasingly possible that a biological relative is, whether for clinical or research purposes or personal interest. The decisions that our relatives make about whether to donate their DNA and medical data for research are also relevant to us. Given that all of us are likely to be confronted with the outcomes from genomic testing within our lifetimes, whether we interact with it as citizens, patients or consumers (Roberts and Middleton, 2018) it is time that the issues linked to genomic medicine are mainstreamed conversationally, or ‘socialised’ (Parry and Middleton 2017).

With more efforts to familiarise publics with genomics, and greater public dialogue about the pertinent issues, risks, and benefits of data donation and sharing, it is possible that those members of the publics who are currently unsure about whether to donate, may make an informed decision to participate. Equally, such dialogue should aim to engage those who are currently unwilling to take part in genomic research, perhaps through discussion of the principles and protections which govern data use. Benefiting from the advances in science is a fundamental human right that all of us have, irrespective of our ethnicity (Knoppers et al., 2014), and future public engagement approaches should consider how to engage with the mistrust reported by certain publics, particularly those who do not self-identify as White.

The size of the sample analysed for this paper does not give us the power to differentiate attitudes between the ethnic groups that are not self-identified ethnically as White. As such, we have deliberately kept our analysis broad. However, we believe that nuances in attitude relevant to ethnicity would benefit from more research, perhaps using a more subtle approach than an online survey can deliver. While this survey has identified potential areas of concern, it is not possible to draw out in greater detail the magnitude of the concerns identified; this would be an important focus of future work. Finally, online surveys have some important limitations, which we have discussed in detail in the methods paper that accompanies our work (Middleton et al., 2018). To understand in detail the reasons why different groups of people are more or less willing to donate or more or less trusting of different research/clinical programmes, there would be value in complementing the current work with a deep qualitative approach.

## 5 Conclusion

To deliver genomic medicine at scale across the world, large datasets containing genomic and phenotypic data are required from millions of people. These datasets are pivotal in variant interpretation to determine whether a result in an individual has been seen frequently in population studies before and whether it is known to be linked to disease. These datasets have traditionally been collected by researchers, scientists and clinicians over the years as genomics has evolved. Yet, fundamentally they rely on people agreeing to donate their data to be used in this way.

The ‘Your DNA Your Say’ survey explores attitudes towards the donation of one's own de-identified personal DNA and medical information to be accessed and shared for research.

In this paper we have reported the profile of public participants from the UK, the USA, Canada and Australia, who say they are unwilling to donate their DNA and medical data. The ‘unwilling’ were more likely to be older, of lower education background, childless, be from the USA or Australia and to identify themselves as an ethnic minority group that is not White. They were also more likely to express concerns that were different to those who were more enthusiastic about data donation, more specifically, they were worried about governments and police knowing information that they had not chosen to share. These findings may reflect persistent concerns about discrimination and persecution. In the future ethical and evidence-based public engagement strategies should consider how to acknowledge and engage with these fears and the cultural and political concerns which accompany potential involvement in genomic research.

## Footnotes

1 Within the survey glossary we explained ‘anonymous’ in more detail: ‘Anonymous: removal of personal information such as name and date of birth. It is questionable as to whether DNA information can ever be truly anonymous as our DNA code is unique to us and thus, in itself, could be used to identify us. However, in the circumstances we are exploring here, by making DNA and medical information 'anonymous', we mean detaching personal identifiers from it.’ What we are actually meaning here is ‘de-identified’ but within the pilot work for the survey we discovered that public participants did not naturally understand this term and ‘anonymous’ was more easily understood, thus we added the glossary definition within the survey itself, to explain this in more detail.
